# Gonococcal Chorioamnionitis with Antepartum Fetal Death In Utero

**DOI:** 10.1155/2015/451247

**Published:** 2015-05-31

**Authors:** B. Coutanceau, J. Boujenah, C. Poncelet

**Affiliations:** ^1^Department of Gynecology and Obstetrics, CHU Jean Verdier, Université Paris XIII, avenue du 14 Juillet, 93 Bondy, Bobigny, France; ^2^Department of Gynecology and Obstetrics, CHI Montreuil, 56 boulevard de la Boissière, 93100 Montreuil, France

## Abstract

We report the case of a patient who developed gonococcal chorioamnionitis resulting in stillbirth at 28 + 4 weeks of pregancy. As this infection is rare and potentially serious, questions remain regarding occurrence and screening for *Neisseria gonorrhoeae* infection.

## 1. Introduction

Chorioamnionitis without membrane rupture is most often due to* Listeria monocytogenes* or* Treponema pallidum*. Maternofetal transmission and neonatal sepsis due to* Neisseria gonorrhoeae* have been reported [1]. These infections usually occur during delivery. However, rare cases of in utero transmission have been published. We report a case of fetal death following gonococcal chorioamnionitis.

## 2. Case Presentation

A 23-year-old Senegalese patient, with two previous instrumental abortions and one vaginal delivery, was admitted at 28 weeks and 4 days with complaints of 38.8°C fever (101.8°F) and uterine contractions.

The patient had history of chronic active hepatitis B. Her living conditions were considered precarious, as she lived in a small apartment with 11 people. The pregnancy, obtained with a stable partner, proceeded normally, with no history of metrorrhagia during first or second trimester. HIV and syphilis screening were negative.

The physical examination revealed maternal tachycardia at 140 beats/min and thin leukorrhea without visible loss of amniotic fluid. Fetal movement had been decreased for three days.

A rapid test for detection of insulin-like growth factor binding protein performed on the vaginal secretions was positive, although no loss of liquid was reported. Fetal ultrasound showed absence of cardiac activity and oligohydramnios, reflecting preterm membrane rupture.

Laboratory tests revealed hyperleukocytosis (24.4 g/L), predominantly neutrophilia, and elevated C-reactive protein (26 mg/L).

A stillborn male fetus weighing 1400 g (90th percentile) was aborted three hours after admission. An intravenous antibiotherapy with amoxicillin (86 mg/kg/day), cefotaxime (43 mg/kg/day), and gentamicin (5 mg/kg/day) was administrated (according to local protocols of our hospital).

Immediate postabortion exam showed a nonmacerated fetus with no apparent birth defects, with the exception of left-hand hexadactyly.

Laboratory exams showed numerous* Neisseria gonorrhoeae* colonies (fluoroquinolone and penicillin resistant, C3G sensitive) in the vagina. Blood cultures were negative.

Anatomopathological examination revealed eutrophic (293 g at the 50th percentile) and congested placenta, with several subchorionic and intervillous thrombi and severe, acute chorioamnionitis associated with funiculitis. No abscess was found. However, bacteriological cotyledon and membranous smears revealed extensive* Neisseria gonorrhoeae* colonies. Fetal autopsy did not reveal any abnormalities. No organ abscess was found.

Clinical evolution was favorable with antibiotherapy. The partner was treated as well. STI screening at three months postpartum was negative for the couple. A follow-up vaginal smear after treatment only revealed* Ureaplasma urealyticum*. A posteriori evaluation of detailed medical history did not mention change of partners and history of chlamydia infection.

## 3. Discussion

*Neisseria gonorrhoeae* infection increased in developed countries since the early 2000s, due to sexual behaviour changes especially in young people. Blatt et al. reported a prevalence of 0.6% in a cohort of 730,796 asymptomatic pregnant women in the United States [2].

The presence of* Neisseria gonorrhoeae* in the female genital tract could have consequences at each trimester of pregnancy. The risk of premature labor is 4 times higher in case of gonococcal cervicitis as reported by Donders [3]. The risk of premature rupture of membranes, chorioamnionitis, and neonatal infection (purulent gonococcal ophthalmia secondary to passage through the infected genital tract, with risk of blindness) seems to be increased. The risk of fetal death in utero could be high, especially without treatment as discussed by MacDonald et al. [4]. Finally, the risk of upper genital tract postpartum infection is increased as well.

Gonococcal chorioamnionitis is rare and cases reported mainly occurred in developing countries [5]. The probable ascending contamination with membranous lesions and the severity of the case challenge the concept of screening for STI before pregnancy.

The current strategy for prevention of gonococcal ocular infection in newborns is based on preventative administration of antibiotic eye drops rather than a strategy of routine screening in pregnant women, which could decrease the occurrence of obstetrical complications.

Though routine screening for chlamydia is recommended in English-speaking countries, screening for gonorrhea is based on an evaluation of risk factors for STI or in populations living in areas with high prevalence of the disease as proposed by the Centers for Disease Control and Prevention [6].

Because of frequent coinfections with chlamydia, screening for gonorrhea in early pregnancy could be proposed in case of a positive test for chlamydia, or in presence of other known risk factors for STI (poor socioeconomic status, young adults, and positive HIV screening).

Severe chorioamnionitis with negative routine bacteriologic screening (group B* Streptococcus*,* Escherichia coli*, and* Listeria monocytogenes*) could raise the issue of gonococcal infection.

## 4. Conclusion

The occurrence of gonococcal infection during pregnancy is rare but at risk. A public health study may evaluate the cost effectiveness of routine or clinically indicated IST screening in preconception care or in the first trimester.

## References

[1] Smith L. G., Summers P. R., Miles R. W., Biswas M. K., Pernoll M. L. (1989). Gonococcal chorioamnionitis associated with sepsis: a case report. *The American Journal of Obstetrics and Gynecology*.

[2] Blatt A. J., Lieberman J. M., Hoover D. R., Kaufman H. W. (2012). Chlamydial and gonococcal testing during pregnancy in the United States. *American Journal of Obstetrics and Gynecology*.

[3] Donders G. (2006). Management of genital infections in pregnant women. *Current Opinion in Infectious Diseases*.

[4] MacDonald N., Mailman T., Desai S. (2008). Gonococcal infections in newborns and in adolescents. *Advances in Experimental Medicine and Biology*.

[5] Yvert F., Frost E., Walter P., Gass R., Ivanoff B. (1985). Prepartal infection of the placenta with *Neisseria gonorrhoeae*. *Genitourinary Medicine*.

[6] Centers for Disease Control and Prevention, Workowski K. A., Berman S. M. (2006). Sexually transmitted diseases treatment guidelines, 2006. *MMWR Recommendations and Reports*.

